# Natural Killer T Cells: An Ecological Evolutionary Developmental Biology Perspective

**DOI:** 10.3389/fimmu.2017.01858

**Published:** 2017-12-22

**Authors:** Amrendra Kumar, Naveenchandra Suryadevara, Timothy M. Hill, Jelena S. Bezbradica, Luc Van Kaer, Sebastian Joyce

**Affiliations:** ^1^Department of Veterans Affairs, Tennessee Valley Healthcare System, Nashville, TN, United States; ^2^Department of Pathology, Microbiology and Immunology, Vanderbilt University Medical Center, Nashville, TN, United States; ^3^Department of Chemistry and Life Science, United States Military Academy, West Point, NY, United States; ^4^The Kennedy Institute of Rheumatology, University of Oxford, Oxford, United Kingdom

**Keywords:** NKT cells, cancer immunotherapy, microbiota, infectious diseases, evolution

## Abstract

Type I natural killer T (NKT) cells are innate-like T lymphocytes that recognize glycolipid antigens presented by the MHC class I-like protein CD1d. Agonistic activation of NKT cells leads to rapid pro-inflammatory and immune modulatory cytokine and chemokine responses. This property of NKT cells, in conjunction with their interactions with antigen-presenting cells, controls downstream innate and adaptive immune responses against cancers and infectious diseases, as well as in several inflammatory disorders. NKT cell properties are acquired during development in the thymus and by interactions with the host microbial consortium in the gut, the nature of which can be influenced by NKT cells. This latter property, together with the role of the host microbiota in cancer therapy, necessitates a new perspective. Hence, this review provides an initial approach to understanding NKT cells from an ecological evolutionary developmental biology (eco-evo-devo) perspective.

## Introduction to Type I NKT Cells

The evolutionary appearance of the vertebrate immune system equipped complex organisms with the ability to resist invasion by pathogenic microbes and to sense and respond to a loss of tissue integrity due to infection, aberrant cell growth, or mechanical injury. As organisms became increasingly more complex and lived beyond their fecund years, a finer ability to discriminate self from non-self was required (1, 2). Thus, the maintenance of homeostasis in such organisms requires the concerted action of multiple cell types that stand poised to respond to a hostile world filled with a seemingly endless array of infectious agents, toxic chemicals, and biologics. The first responders in this elaborate defensive network have historically been classified as members of the more archaic, multi-modular innate immune system. Should the innate defenses prove insufficient, the evolutionarily younger, adaptive immune system—consisting of B and T lymphocytes—is recruited to restore the homeostatic state. The quick-acting cells of the innate immune system senses an altered homeostatic state with pattern recognition receptors to detect conserved molecular structures shared by many pathogens alike (3, 4). By contrast, the slow-responding, adaptive immune system senses alterations in homeostasis by using diverse, clonally distributed B cell receptors (BCR and their secreted counterparts, antibodies), and T cell receptors (TCRs), respectively.

Bridging the gap between innate and adaptive immune responses are the innate-like B and T lymphocytes. These are a group of cells that express a relatively restricted repertoire of receptors generated through somatic recombination, yet unlike conventional T and B cells, exhibit innate-like recognition principles and functional responses (5). Innate-like lymphocytes include both T cells (γδ T cells, natural killer T cells, mucosal-associated invariant T lymphocytes, and CD8αα-expressing intestinal intraepithelial lymphocytes) and B cells (B-1 B cells and marginal zone B cells). The evolutionary appearance of this group of immune cells, including natural killer T cells (NKT cells) endowed upon vertebrates the capacity to initiate and amplify both the innate and adaptive immune responses. By virtue of their immunoregualtory functions, innate-like lymphocytes can fine-tune the nature and magnitude of these immune responses (6). Although each immune module plays a specific role, it is the controlled integration of multiple modules that results in an effective inflammatory response that is essential in maintaining a stable *milieu intérieur* (7).

NKT cells—originally defined as cells that co-express key natural killer (NK) cell surface markers and a conserved αβ TCR repertoire—are thymus-derived, innate-like T lymphocytes. The functions of NKT cells are controlled by self and non-self-lipid agonists presented by CD1d molecules (8). The majority of NKT cells (type I, invariant NKT) express an invariant TCR α-chain (Vα14Jα18 in mice; Vα24Jα18 in humans). The invariant α-chain pairs predominantly with Vβ8.2, Vβ7, or Vβ2 in mouse NKT cells, or Vβ11 almost exclusively in human NKT cells. A small NKT cell population—referred to as type II NKT cells—expresses a more diverse TCR repertoire and recognizes a distinct group of lipid antigens; these, however, are the focus of other reviews (9–14). The recognition of lipid agonists rapidly activates NKT cells, which respond just as quickly by secreting a variety of cytokines and chemokines, and upregulate costimulatory molecules. By acting promptly, NKT cells alert and regulate the effector functions of myeloid and lymphoid cells. In so doing, NKT cells play a critical role in controlling microbial and tumor immunity as well as autoimmune and inflammatory diseases (6, 15–17).

## Multiple Mechanisms Activate NKT Cell

The functions of NKT cells are controlled by CD1d molecules. CD1d molecules bind to and present a variety of lipid ligands to reactive T cells (18). Numerous *in vitro* and *in vivo* studies using the synthetic lipid α-galactosylceramide (αGalCer, KRN7000) and its analogs (Table 1 and references therein) has led to our current understanding of NKT cell biology. αGalCer is a natural product isolated from the marine sponge, *A. mauritianus*. The gut bacterium, *Bacteroides fragilis*, and the fungus, *Aspergillus fumigatus*, also biosynthesise αGalCers and/or related compounds (Table 1 and references therein). Hence, αGalCer and related compounds may be more prevalent in nature than previously thought and the NKT cell biology so gleaned may be highly relevant.

**Table 1:** Synthetic, microbial, and self NKT cell agonists—structures and properties.

Lipid (class)	Chain Length^a^	Structure	Agonist^b^	Reference
αGalCer (GSL)	C18; C24:1	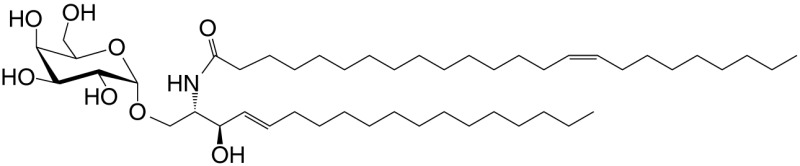	IFN-γ, IL-4 self	(19)
Agel 9b (GSL)	C17 (C_16_-Me); phyto C24	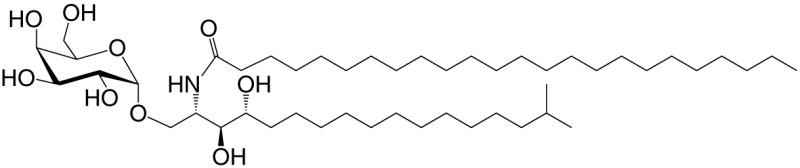	Anti-tumor; *Agelas mauritianus*	(20, 21)
KRN7000 αGalCer (GSL)	C18-phyto; C26	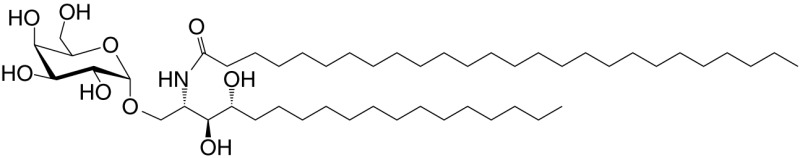	Very strong; robust IFN-γ IL-4 and other cytokines; synthetic analog of Agel 9b	(22)
α*C*Gal-Cer (GSL)	C18-phyto; C26	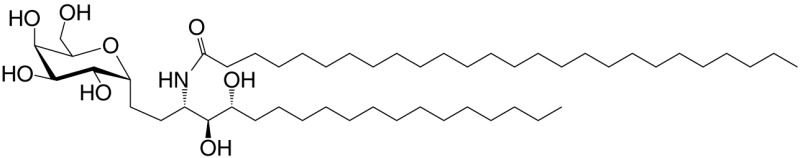	Weak (mo)-to-none (hu); IFN-γ; synthetic	(23)
OCH (GSL)	C9-phyto; C24	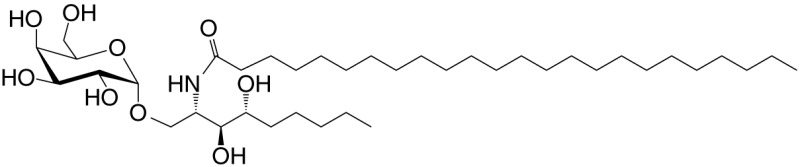	Weak (mo)-to-none (hu); IL-4 (low-to-no IFN-γ); synthetic	(24)
C20-diene (GSL)	C18-phyto; C20:2	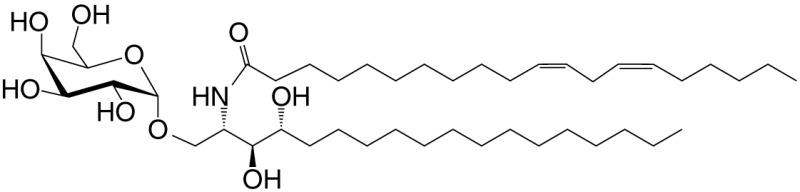	Strong; IL-4 (low-to-no IFN-γ); synthetic	(25)
αGalCer (GSL)	C17-C_3_OH; C17	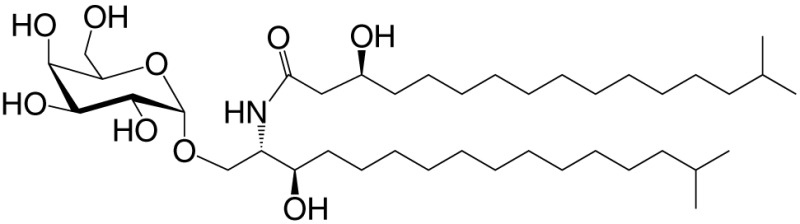	Stimulatory and inhibitory *Bacteroides fragilis*	(26, 27)
αGalU Cer (GSL)	C18-phyto; C14	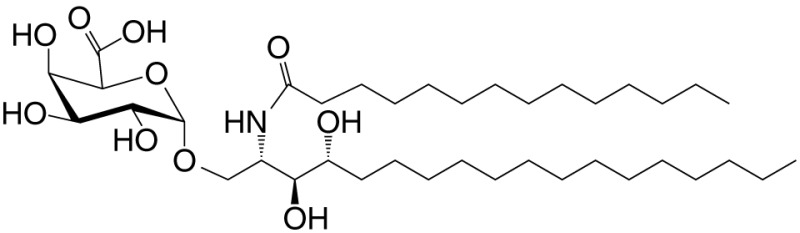	Weak; *Sphingomonas* spp.	(28–30)
Asp B (GSL)	C20:2-C_9_ Me; C16-C_2_ OH	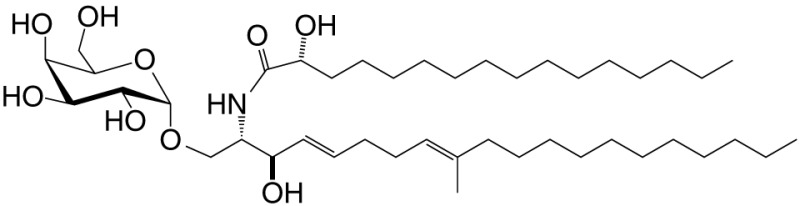	Weak; *Aspergillus fumigatus*	(31)
αGlc-6-acyl-Chol	C14	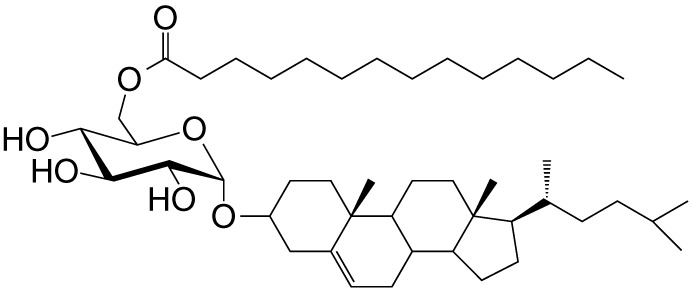	Strong; binds a small NKT cell subset (mo); *Helicobacter pylori*	(32)
βGalCer (GSL)	C18; C24:1	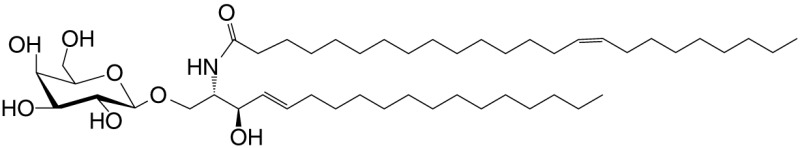	Weak; self	(33, 34)
iGb3 (GSL)	C18; C24	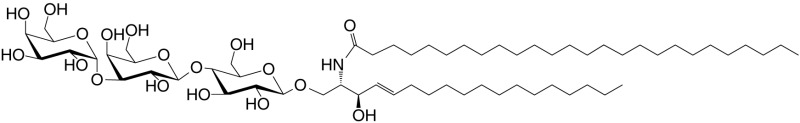	Weak (mo)-to-none (hu); self	(35)
αGal-DAG (GGL)	*sn1*-C18:1; *sn2*-C16	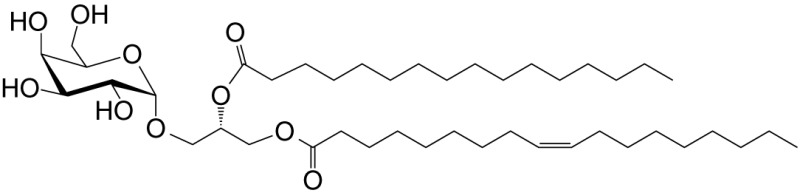	Weak (mo)-to-none (hu); *Borrelia burgdorferi*	(36)
αGlc-DAG (GGL)	*sn1*-C18:1; *sn2*-C16	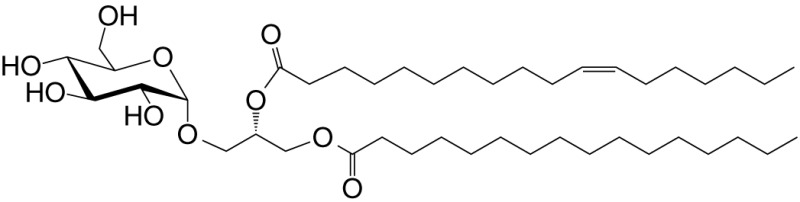	Weak; *Streptococcus pneumoniae*	(37)
PtdIno (GPL)	*sn1*-C18:1; *sn2*-C18:1	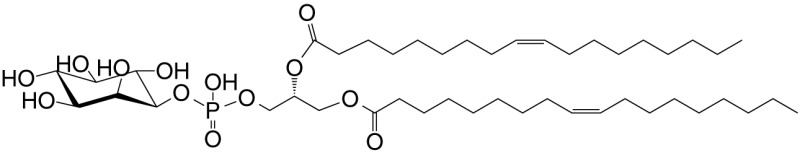	Week (mo)-to-no (hu); self	(38, 39)
Plasma-logen (GPL)	*sn1*-C16 vinyl-ether; *sn2*-lyso	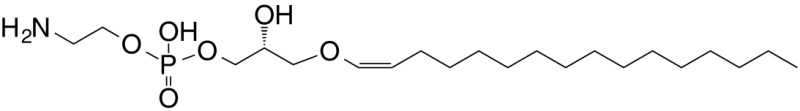	Positive selection (mo); self	(40)
Lyso-PtdCho (GPL)	*sn1*-C16; *sn2*-lyso	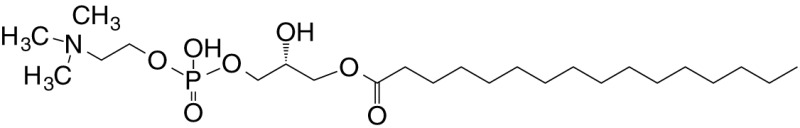	Weak (hu)-to-none (mo); GM-CSF (no IL-4, IFN-γ); self	(41)

*This table is adapted from Ref. (8, 42)*.

*Agel, agelasphin; Asp B, asparamide B; Chol, cholesterol; DAG, diacylglycerol; GalCer, galactosylceramide; GalUCer, galacturonosylceramide; GlcCer, glucosylceramide; PtdCho, phosphatidylcholine; PtdIno, phosphatidylinositol; sn, stereo nomenclature for glycerolipids; GGL, glycoglycerolipid; GPL, glycerophospholipid; GSL, glycosphingolipid; mo, mouse; hu, human*.

*^a^Sphingosine/phytosphingosine chain length indicated first and N-acyl chain length second*.

*^b^Agonist strength based on Ref. (43)*.

**Figure 1:**
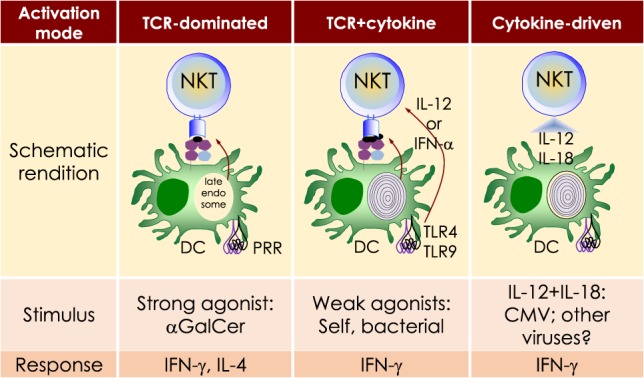
Three distinct strategies activate mouse NKT cells. Potent NKT cell agonists—such as αGalCer—directly activate NKT cells without the need for a second signal, in a T cell receptor (TCR)-signaling dominated fashion (left panel). Alternatively, microbes containing toll-like receptor (TLR) ligands such as LPS activate NKT cells by inducing IL-12 production by DCs, which amplifies weak responses elicited upon the recognition of CD1d bound with self-glycolipids by the NKTCR. Several endogenous lipid agonists have been identified and characterized (see Table 1). Some microbes such as *Sphingomonas capsulata*, which are α-Proteobacteria, synthesize α-anomeric glycolipids for their cell walls. These glycolipids, when presented by CD1d, weakly activate NKT cells directly. In the presence of a second signal—generally a pro-inflammatory cytokine such as IL-12—such weak agonists strongly activate NKT cells (middle panel). Intriguingly, NKT cells can be activated solely by cytokines—mainly IL-12— in a TCR-independent manner (right panel). This diagram rendering the different strategies to NKT cells is an adaptation of past reviews (8, 44) and is based on works cited in the text.

αGalCer is a potent NKT cell agonist, which when presented by CD1d molecules directly activates NKT cells in a TCR-dependent manner without need for additional signals. This activation mechanism is considered TCR-dominated mode of NKT cell activation (Figure 1).

*Sphingomonas* spp. biosynthesises an αGalCer-related compound, α-galacturonosylceramide (αGalACer). Other weak NKT cell agonists include microbial glycosphingolipid [GSL; e.g., αGalCer-related asparamide B (*A fumigatus*)], diacylglycerolipids [e.g., α-galactosyl- (*Borrelia burgdorferi*—the agent of Lyme disease) and α-glucosyl-diacylglycerol (*Streptococcus pneumoniae*)] and cholesteryl-α-glycoside [e.g., cholesteryl-6-O-acyl α-glucoside (*Helicobacter pylori*)] (Table 1 and references therein). Being a weak agonist, NKT cell activation by these microbial glycolipids requires a second activation signal from inflammatory cytokines. Such inflammatory cytokines result from dendritic cells (DCs) that are activated through their pattern recognition receptors (45–47). This activation mechanism is considered TCR- and cytokine-mediated mode of NKT cell activation (Figure 1)—a feature that is important for NKT cell activation by weak microbial and self-lipid agonists.

NKT cells react to CD1d molecules presenting self-lipids on host APCs in the presence of a second signal (6, 48). The inability to activate NKT cell hybridomas by using artificial APCs lacking βGlcCer synthase (49) and impaired NKT cell development in mice lacking βGlcCer synthase in their thymocytes (50), suggested that a cellular, βGlcCer-derived GSL is an endogenous mouse NKT cell agonist (49, 50). Several microbes—bacteria (e.g., *Staphylococcus aureus, Salmonella typhimurium, Listeria monocytogenes*, etc.), fungi (e.g., *A. fumigatus*) and viruses—activate NKT cells but do not biosynthesise NKT cell agonists. Such microbes induce the biosynthesis and/or presentation of self-lipids, which are thought to be mammalian αGalCer and perhaps iGb3 (19, 28, 35). As self-lipids are weak NKT cell agonists, NKT cell activation is bolstered by IL-12 secreted by DCs activated through dectin-1 DCs (31, 47) or toll-like receptor (TLR)-4 (45, 46). This activation mechanism is a variation on the TCR- and cytokine-mediated mode of NKT cell activation and a feature that is important for NKT cell activation by microbes that do not themselves biosynthesise an NKT cell agonist.

Type I interferon (IFN)—produced by DCs activated by the TLR9 ligand CpG—can serve as a second signal for NKT cell activation in conjunction with the presentation of sialylated cellular glycolipids by CD1d molecules (51). This finding is significant because almost all viral infections induce type I IFN response. Even though viruses do not biosynthesise NKT cell agonists, or any lipid for that matter, viral infections also activate NKT cells (52–62). Perchance, in such a circumstance, NKT cell activation occurs *via* the recognition of a self-lipid(s) presented by CD1d in the presence of inflammatory signals relayed by type I IFNs.

NKT cells are activated by the combined actions of IL-12 and IL-18. Under such conditions, NKT cell activation does not require the recognition of a CD1d-restricted agonist (63–65). This latter mechanism is considered cytokine-driven NKT cell activation (Figure 1). This mechanism is important for immunity to cytomegalovirus (65). Summarily, these multiple modes of activation suggest that NKT cells have evolved many different mechanisms to sense an altered homeostatic state caused by microbial infections. How activated NKT cells steer downstream innate and adaptive immune responses is described below.

## Transactivation of Innate and Adaptive Immune Responses by Activated NKT Cells

NKT cells form immune synapses upon recognition of lipid agonists presented by CD1d molecules displayed on APCs or planar membranes. The kinetics NKTCR/ligand interactions determine the functional outcome (66). Positive cooperative engagement of CD1d-lipid agonistic complexes by the NKTCR allows NKT cells to recognize subtle changes in cellular lipid content and to actuate a response (67). Upon activation, NKT cells rapidly polarize IFN-γ and lytic granules to the immune synapse to transmit an effector response (66, 68, 69). The synaptic transmission of effector molecules controls downstream innate and adaptive immune responses as described below.

**Figure 2:**
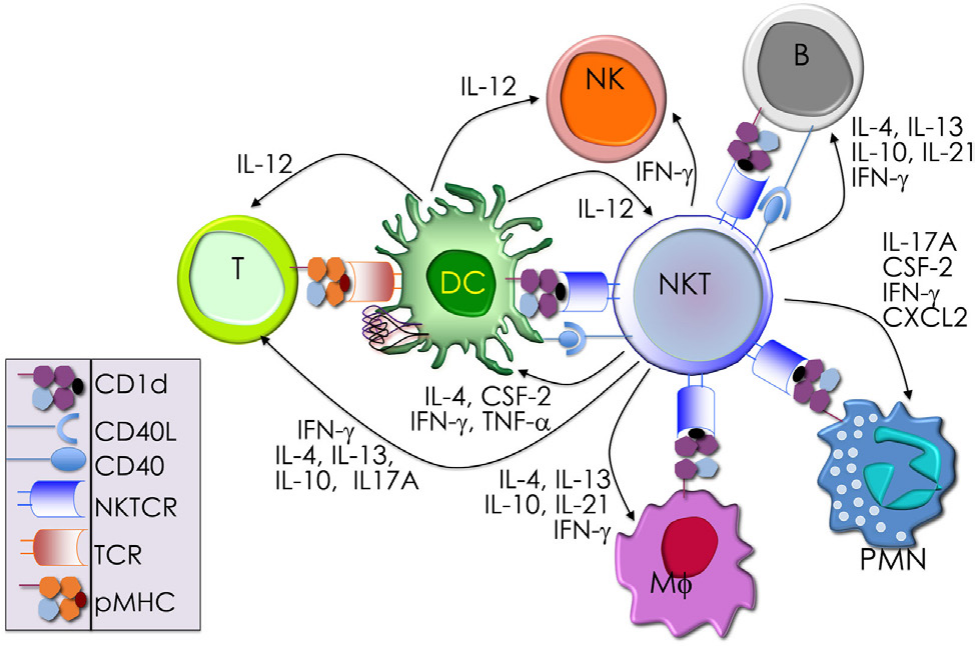
The immunological effector functions of mouse NKT cells. The interactions between the invariant natural killer T (NKT) cell receptor and its cognate antigen, as well as interactions between costimulatory molecules CD28 and CD40 and their cognate ligands CD80/86 (B7.1/7.2) and CD40L, respectively, activate NKT cells. Activated NKT cells participate in crosstalk with members of the innate and the adaptive immune systems by deploying cytokine and chemokine messengers. Upon activation *in vivo*, NKT cells rapidly secrete a variety of cytokines and chemokines, which influence the polarization of CD4^+^ T cells toward T helper (Th)1 or Th2 cells as well as the differentiation of precursor CD8^+^ T cells to effector lymphocytes, and B cells to antibody-secreting plasma cells. Some of these mediators facilitate the recruitment, activation and differentiation of macrophages and dendritic cells (DCs), which results in the production of interleukin (IL)-12 and possibly other factors. Interleukin (IL)-12, in turn, stimulates NK cells to secrete interferon (IFN)-γ. Thus, activated NKT cells have the potential to enhance as well as temper the immune response. This schematic rendition of NKT cell effector functions is an adaptation of past reviews (6, 8, 44, 70) and is based on works cited in the text.

Akin to the cells of the innate immune system (e.g., neutrophils, Mϕ, DCs, and NK cells), NKT cells respond within the first several hours of agonist recognition and secrete copious amounts of effector cytokines and chemokines (Figure 2). The nature of the activating NKT cell agonist controls the nature of the cytokine response (see Table 1). For example, the synthetic agonist αGalCer, within 30–90 min, elicits a wide variety of cytokines (Figure 2). Nonetheless, αGalCer variants containing different lipid chain length or unsaturation typically induce an IL-4 cytokine response (24, 25). By contrast, other αGalCer variants that have an altered glycosidic linkage, a chemically modified acyl-chain, or a modified sphingoid base, potently induce an IFN-γ response (Table 1 and references therein). Thus, it is possible to steer desirable immune responses against cancers by harnessing lipid agonists that induce therapeutic cytokine responses. This feature of αGalCer variants is further accentuated by the ability of activated NKT cell responses to transactivate cells of the innate and adaptive immune systems as narrated briefly below (see Figure 2).

Dendritic cells, especially CD8α^+^ DCs, which are a major producer of IL-12 (71), play a critical role in glycolipid agonist presentation and NKT cell activation (72–78). Activated NKT cells reciprocate by activating the interacting DCs. DCs so activated rapidly mature. Hence, they upregulate costimulatory molecules CD40, CD80, and CD86; several molecules critical for protein antigen capture and peptide presentation, such as DEC205 and MHC class II molecules (79); and induce the production of IFN-γ, tumor necrosis factor (TNF)-α, and IL-12 (80–83). IFN-γ produced by activated NKT cells coupled with CD154 (CD40 ligand on NKT cells) and CD40 (on DCs) mediate the NKT-DC crosstalk (81, 84). This crosstalk steers multiple downstream immune responses: (1) the number and phenotype of DCs after tumor induction (85). (2) IL-12 and IL-18 resulting from NKT-DC crosstalk transactivates NK cells to produce IFN-γ (82). (3) NKT-DC crosstalk can result in IL-4, IL-6, IL-13, and IL-21, which together can enhance B cells responses to protein antigens by B cells (86–93). (4) NKT-DC cross talk licenses DCs for antigen cross-presentation to CD8^+^ T cells (94–96), and the activation and differentiation of CD4 and CD8 T cells (79, 95–97). Through these bidirectional interactions, NKT cells and DCs cooperate to amplify and direct both innate and adaptive immune responses. Hence, NKT cells are an attractive target for cancer immunotherapies (98–102).

## Implications for Cancer Immunotherapy

NKT cells have long represented an attractive target for tumor immunotherapy (103, 104). Numerous studies in both humans and mice have demonstrated their ability to directly target CD1d-expressing tumor cells (105–108), recruit and activate anti-tumor effector cells of the innate and adaptive immune systems (100, 109–114), and control the activity of immunosuppressive cells in the tumor microenvironment. After *in vivo* administration of αGalCer, NKT-DC cross-talk-mediated NK cell activation results in IFN-γ response (82) and, potentially, the anti-tumor effect of αGalCer (85, 115).

The potent anti-metastatic activity of αGalCer in mice (20, 116), which is NKT cell mediated (22), prompted investigations in the role of NKT cells in natural immunity against tumors. Such investigations include chemically induced tumors, transplanted tumors, and tumors arising in genetically engineered animals (115). The outcomes of these studies have been promising because NKT cells exhibit natural immunity against different cancer models. Independent studies have sometimes reported conflicting results as to the importance of NKT cells in the anti-tumor response, particularly with carcinomas induced by the topical carcinogen methylcholanthrene (117, 118). Such conflicting results were likely due to unknown environmental and/or genetic factors present in the mice used as controls in similar experiments by different groups (117). Studies in mice revealed that αGalCer variants that induce type I inflammatory response (see Table 1) were protective against tumor metastases. The mechanistic basis of this anti-metastatic effect remains elusive. Nonetheless, the ability of NKT cells activated by αGalCer variants to steer desirable downstream effector functions, such as NK cells, cytotoxic T cells, Th1 and Th17 cells, γδ T cells, IFN-γ, and direct lysis of myeloid lineage cells may underlie the outcome (100, 115). The anti-tumor activities of NKT cell agonists have already been exploited in a variety of clinical trials. The outcomes of these trials have also been promising (103, 104, 119–121).

### Genomic Control of NKT Cell Development

**Figure 3:**
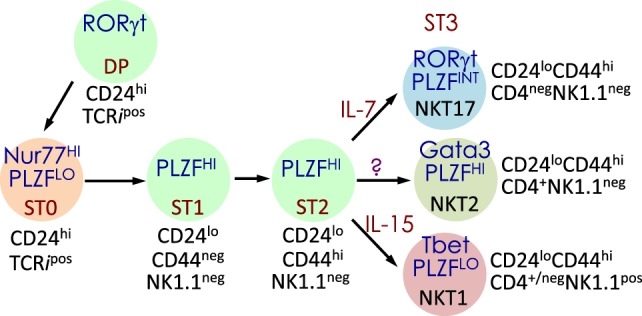
Schematic rendition of mouse NKT cell developmental stages: precursor ST0, immature ST1 and ST2 and mature ST3 and NKT1, 2, and 17 are functional subsets. Early developmental steps are common to both NKT cell and conventional T cell lineages as commitment to the NKT lineage occurs at the CD4 and CD8 double-positive (DP) stage. NKT cell ontogeny begins with rearrangement of the *V*α*14* to *J*α*18* T cell receptor (TCR) α-chain gene segments and after its interaction with the positively selecting CD1d-self-lipid complex. Stage-specific NKT cell markers—e.g., CD24, CD44, and NK1.1—and subset-specific differentiation signals and transcription factors are indicated. Interleukin (IL)-7 and IL-15 are cytokines that mediate intercellular communication. NKTCR signaling turns-on the master transcription factor promyelocytic leukemia zinc finger (PLZF), which controls multiple molecular events that distinguish NKT cells from all of the other thymus-derived T lymphocytes. Additional molecular cues include Fyn and Lck, which are Src (cellular protein homologous to the *Rous sarcoma virus* oncogene) kinases (protein phosphorylation enzymes) essential for transmitting NKTCR signals from the plasma membrane to inside of the cell. Fyn also transmits signals relayed from SLAM (signaling lymphocyte activation molecule) through the adapter protein SAP (SLAM-associated protein). Protein kinase C (PKC)-θ processes NKTCR signaling and activates the transcription factor nuclear factor-κB (NF-κB). Other transcription factors, such as Egr-2, Ets-1, GATA3, Id2, Id3, MEF, Nur77, RORγt, and T-bet, some of which are also essential for functional differentiation of NKT cell subsets (refer to Figures 4 and 6) and act at distinct stages of NKT cell development. This diagrammatic rendition of NKT cell development is an adaptation of a past review (8) and is based on works cited in the text.

NKT cells development and maturation occurs in the thymus (122, 123). Thus, genetically altered mice in which thymocytes do not develop beyond the double-negative (DN)2/DN3 stage also fail to develop NK1.1^+^ T cells (124). [Note: historically, prior to the development of CD1d-lipid tetramers (125, 126), NKT cells were identified by co-expression of the NK1.1 marker and a TCR. Hence, in pre-tetramer literature, they were referred to as NK1.1^+^ T cells (127).] Thymic NK1.1^−^ NKT cells were later recognized as a CD1d tetramer^+^ NK1.1^−^ subset that precedes NK1.1^+^ NKT cells in development (128, 129). Current literature refers to the IFN-γ-producing, mature, stage 3 (st3) NKT cells as NK1.1^+^ NKT cells (Figure 3). Furthermore, NKT cells do not develop in mice harboring mutations in genes (e.g., *Myb*, that encodes the transcription factor c-Myb, *Rorc*, which encodes RORγt, and *Tcf12* that codes for HEB) that impair survival of immature double-positive (DP) thymocytes—cells that co-express both CD4 and CD8 co-receptors— (130–133). Moreover, *V*α*14* and *J*α*18* rearrangement occurs at a late DP stage (130, 132). Consistent with this finding, NKT cells develop in NKT cell-deficient *J*α*18*-deficient (Ja18^−/−^) mice that receive highly purified tetramer-negative, DP-high thymocytes (134). These findings together support the notion that commitment to the NKT cell lineage occurs at the DP stage much alike conventional T cells (135). That notwithstanding, compelling new data indicate that *V*α*14* and *J*α*18* rearrangement can occur within CD4- and CD8-negative (DN) thymocytes. Additional data indicate that a fraction (~15%) of NKT cells that differentiate into NKT1 cells emerge from DN thymocytes (136). Hence, an alternative precursor can give rise to functional NKT cells.

Positive selection of NK1.1^+^ T cells depends on DP thymocytes (122). Developing NKT cell-DP thymocyte interactions involve both self-lipid-bound CD1d/NKTCR (22, 116, 137–139) and signaling lymphocytic activation molecule (SLAM)–SLAM interactions (140–142). These interactions are critical to NKT cell maturation, which involves protein kinase Cθ-NF-κB (143) and NFAT-Egr2 (144–146) activation downstream of the NKTCR, and SLAM-associated protein-Fyn activation downstream of SLAM (140, 141, 147, 148). Signals so transmitted from the cell surface are relayed through multiple signaling nodes in the cytoplasm and integrated in the nucleus into a unique transcriptional program (Figure 3). A key nuclear event involves the activation of the zinc finger BTB domain-containing-16 (*Zbtb16*) gene that codes for promyelocytic leukemia zinc finger (PLZF). The PLZF-mediated genomic control distinguishes the unique NKT cell functions from those of the other T lymphocytes (149, 150). NK1.1^−^ NKT cells undergo several rounds of cell division, retaining some in the thymus with the remaining emigrating and populating the peripheral lymphatic organs. Thence, NK1.1^−^ NKT cells mature to become NK1.1^+^ NKT cells, both in the thymus and the periphery (Figure 3). A key feature of this maturation process is the acquisition of cytokine secretion function in a less well-understood mechanism (148) and the differentiation into three functional subsets: NKT1, NKT2, and NKT17 (discussed below). These NKT cell subsets marked by the same subset-specific transcription factors and cell surface markers expressed by the corresponding T helper cell subsets (151–156).

**Figure 4:**
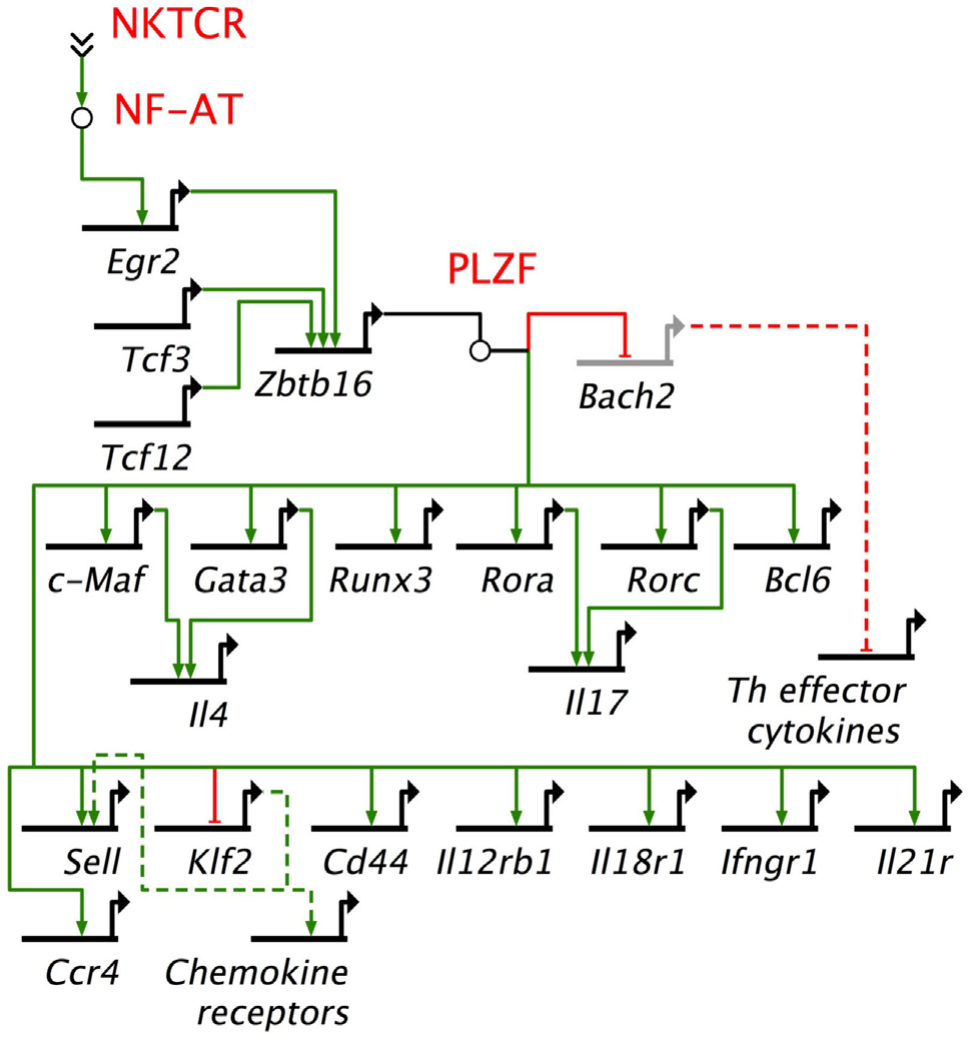
Promyelocytic leukemia zinc finger (PLZF)-driven gene regulatory network directs innate effector function in mouse NKT cells. NKTCR stimulation by self-glycolipid antigen activates downstream nuclear factor of activated T cells (NF-AT), which activates *Egr2* gene. Egr2 is essential for *Zbtb16* gene expression downstream of NKTCR stimulation. PLZF encoded by *Zbtb16* activates T helper (Th) lineage-specific transcription factors, except *Tbx21*, which codes for the Th1 master regulator Tbet. Promyelocytic leukemia zinc finger (PLZF) also binds to multiple *cis*-regulatory elements to repress *Bach2*, which is a repressor of Th cytokine genes. In addition, PLZF binds to *cis*-regulatory elements of a variety of cytokine and chemokine receptor genes. *NF-AT, Tcf3, Tcf12, Egr2, Zbtb16, Bach2, c-Maf, Gata 3, Runx3, Rora, Rorc, Bcl6*, and *Klf2* encode transcription factors. Other genes under PLZF control encode effector proteins, mostly cytokines (e.g., *Il4*), cytokine receptor (*Il12Rb1, Ifngr1, Il21r*), chemockine (*Ccr4*) or cell adhesins [*Cd44, Sell* (L-selectin)]. *Bach2* represses the induction of T helper (Th) effector cytokine genes. Thick black lines, *cis*-regulatory elements of genes; solid green lines, enhancer; solid red lines, repressor; dashed green lines, indirect evidence for enhancement; dashed red lines, indirect evidence for repression. Based on Ref. (145, 149, 150, 158).

Gene regulatory networks (GRNs) are composed of *trans*-regulatory factors—generally made up of transcription factors and regulatory RNA such as microRNAs and long non-coding RNA—and *cis*-regulatory regions generally found upstream of genes whereupon transcription factors bind to control lineage-specific gene expression. GRNs unveil the origins and evolution of cell lineages (157). Many transcription factors have been studied in relation to NKT cell development and function. Among these, PLZF works as a master transcription factor controlling the development of innate-like functions within NKT cells (Figure 4) (149, 150, 158). Mice harboring a loss-of-function PLZF mutation or lacking PLZF demonstrated poor NKT cell development, and those NKT cells that developed were NK1.1^−^ and homed to lymph nodes but not to tissues such as thymus and liver where they are found abundantly in wild type (wt) mice (149, 150). Additional studies indicated that PLZF binds to *cis* elements of effector cytokine and homing receptor genes to direct their expression within NKT cells (Figure 4) (158). Furthermore, forced expression of a *Zbtb16* transgene in all T cells during thymic development resulted in the acquisition of an innate-like phenotype and function in conventional T cells (158). These findings heralded PLZF as a lineage-specific master regulator of transcription (149, 150, 158), and has led to the unveiling of a GRN that controls effector differentiation in developing NKT cells (Figure 4).

The induction of *Zbtb16* is controlled in part by acetylated Egr2 (159), which is induced downstream of NKTCR signaling (144). A recent study demonstrated that the gene encoding the histone acetylase GCN (general control non-derepressible) 5 acetylates a critical lysine residue in Egr2. DP thymocyte-specific depletion of GCN5 blocked the progression of NKT cell development from stage 0 to stage 1 in a cell intrinsic manner. This stage 0 to stage 1 developmental block was due to transcriptional downregulation of the lineage driving gene *Zbtb16* and other genes such as *Runx1, Tbx21*, and *Il2rb* that are essential for proper NKT cell development (159). GCN5 itself is an acetylated protein. Whether its function during NKT cell development depends on acetylation is currently unknown. In some models, the function of GCN5 depends on its deactylation (160). Should GCN5 function in NKT cells depend on deacetylation, whether and which sirtuins [silent mating type information regulation 2 homologs 1–7 (160)] play this role in NKT cells remains to be established.

Even though the mouse invariant Vα14i TCR α-chain has the potential to pair with virtually all available TCR β-chains, the peripheral NKT cell repertoire consists of Vα14i paired with a restricted set of β-chains, viz., Vβ8, Vβ7, and Vβ2 (161). There are two views to the events that sculpt this semi-invariant NKTCR repertoire: the predominant view is that such a semi-invariant NKTCR repertoire is built exclusively by positive selection (162). The competing hypothesis—that both positive and negative selections sculpt the semi-invariant NKTCR repertoire—is supported by indirect evidence (163–166).

Two lines of evidence support the notion that positive selection sculpts the NKT cell repertoire. CD1d molecules have a recycling motif in their cytoplasmic tail, which is essential for the endo/lysosomal exchange of CD1d-bound lipids and their subsequent presentation to NKT cells. Transgenic mice expressing a mutant CD1d molecule that has lost the ability to recycle do not develop NKT cells, suggesting that positive selection requires a recycling CD1d molecule (167). Another line of support comes from the study of CD1d-null mice, which contain a small number of CD1d-tetramer^+^ thymocytes. These pre-selection thymocytes also express only the Vβ8, Vβ7, and Vβ2 β-chains expressed by the post-selection NKT cells. Such pre-selection thymocytes expand the same NKTCR repertoire when stimulated with a putative self-glycolipid called isogloboside-3 *in vitro* (35, 161). These lines of evidence support positive selection as the sole model for sculpting the NKT cell repertoire.

Deletion of the gene coding for NKAP (NF-κB activating protein) in DP thymocytes specifically blocks the development of NKT cells but not conventional T cells (168). NKAP colludes with HDAC3 (histone deacetylase 3) to function as a transcriptional repressor (169). Accordingly, deletion of the *Hdac3* gene in DP thymocytes completely blocks NKT cell development, while conventional T cell development proceeds normally (168). Hence, the repression of target genes at the DP thymocytes stage by the combined action of NKAP and HDAC3 is essential for positive selection of the NKT cell lineage.

Three lines of evidence support a potential role for negative selection in pruning self-reactive NKT cells for sculpting a functional repertoire: first, all available TCR β-chains can pair with the Vα14i TCR α-chain and react with CD1d tetramer, yet only Vβ8, Vβ7, and Vβ2 β-chains are expressed by the post-selection NKT cells (161). This finding can be explained only by negative selection of the majority of the β-chains and not by the failure to survive owing to the inability to interact with CD1d or to failed positive selection (38, 161). Second, transgenic over expression of either mouse or human CD1d in DP thymocytes and thymic myeloid cells results in fewer NKT cells and, those that remain, display altered Vβ usage (163, 170). Furthermore, only wt 16.5-day post-coitus mouse fetal thymic organ cultures (FTOCs), but not FTOCs from CD1d-overexpressing transgenic animals, fostered NKT cell development (163). Finally, exogenous addition of αGalCer, to wt mouse FTOCs resulted in NKT cell depletion (163, 164). Likewise, *in vivo* αGalCer injections into neonatal mice also resulted in the intra-thymic depletion of NKT cells (164). Together, these findings provide compelling evidence, albeit indirect, supporting a role for negative selection in sculpting a functional NKT cell repertoire.

Agonistic ligand(s)—those that positively select in the thymus being similar or identical to ligands that activate in the periphery (19, 27, 171)—selects NKT cells, which strikingly contrast antagonist ligand-mediated selection of conventional T cells. Further, SLAM–SLAM interactions, which activate PKC-θ *via* the SAP-FynT signaling module, mediate persistent interactions between developing NKT cells and the selecting DP cells (140, 141, 147, 172–175). NF-κB provides a survival signal to escape death that could result from these high affinity interactions (166, 176–182). Current evidence suggests that signals relayed through the TCR–PKCθ–CARMA1 axis are integrated by NF-κB to prevent death of developing NKT cells (143, 166, 183). But the signals relayed by the TCR-PKCθ-CARMA1 axis only partially accounts for such death signals. Consistent with this conclusion is the finding that TNF-α ligation of TNF receptor superfamily member 1a (TNFR1) relays caspase 8 and caspase 9 activation signals to mediate NKT cell death. This death signal is also obviated by NF-κB activation (183). Additional signals also mediate NKT cell survival during development (181, 184–192). Hence, escaping cell death from multiple signals may be a key feature of thymic NKT cell development. Whether this cell death is the basis for negative selection of NKT cells currently remains unknown.

NKT cells must tightly regulate NF-κB activation as mice that lack RelA or cannot activate NF-κB poorly develop NKT cells (143, 176, 177). On the other hand, mice that express overactive NF-κB or lack the negative regulator of NF-κB signaling CYLD, develop NKT cells but fail to mature and populate the lymphoid organs and peripheral tissues (181). Hence, NF-κB may function as a rheostat to set the threshold for peripheral NKT cell activation. Such a threshold may be critical as their selection and function are controlled by agonistic ligand(s) so as to prevent autoreactivity. How NF-κB functions as a rheostat in developing NKT cells needs elucidation.

### NKT Cell Subsets, Frequency Variation, and Microbial Influences on Function: An Ecological Perspective

Recent findings on NKT cell developmental properties may be best understood from an ecological perspective. These properties include, (a) functional NKT cell subsets and the division of labor; (b) NKT cell frequency variation; (c) tissue environment-dependent NKT cell subset frequency variation; and (d) gut microbiota-dependent peripheral NKT cell maturation and reciprocal NKT cell control over gut microbiota.

## Functional NKT Cell Subsets and the Division of Labour

**Figure 5:**
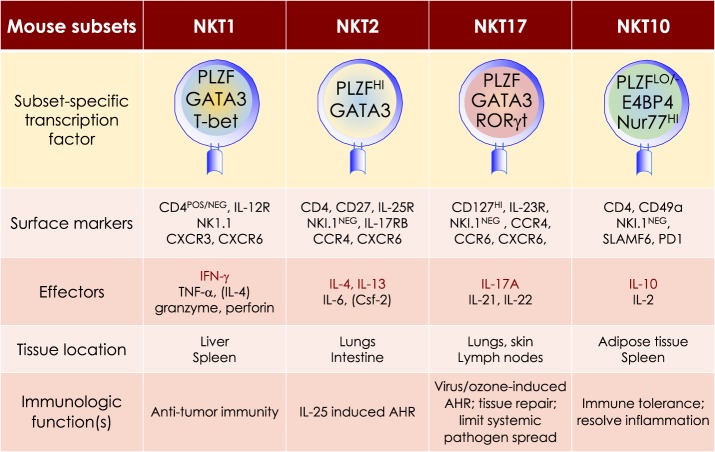
At least four mouse NKT cell subsets divide-up the labor. The four NKT cell subsets reflect T helper (Th)1, Th2, Th17, and Treg subsets. The transcription factors and the prototypic effector molecules as well as the locations and functions, which define the four NKT cell subsets, are represented. The text contains detailed description of each subset. This diagrammatic rendition of NKT cell subsets is an adaptation of a past review (8) and is based on works cited in the text.

NKT cell activation results in rapid secretion of pro-inflammatory and regulatory cytokines and chemokines. This property in conjunction with the capacity to transactivate a variety of innate and adaptive immune cells—see subsection on Transactivation—allows NKT cells to steer downstream immune responses. NKT cells are heterogeneous, consisting of at least four distinct subsets—NKT1, NKT2, NKT10, and NKT17. In addition, at least one induced subset, NKTfh, is also recognized. As with conventional CD4^+^ T cell subsets, NKT cell subsets are characterized by prototypic cytokine responses and subset-specific transcription factors (Figure 5). Each subset is represented at different proportions in various mouse strains (151–155).

MOUSE NKT1 CELLS are marked by either the expression of CD4 or the absence of CD4/CD8 co-receptors. NKT1 cell activation results in a Th1-like cytokine response. The majority of mouse splenic and hepatic NKT cells are NKT1 subset, especially in the C57Bl/6 strain. NKT1 cell differentiation depends on T-bet (*Tbx21*) and IL-15 but less on GATA3 (151, 152, 187, 189–191). Unlike HDAC3 depletion in DP thymocytes, NKT cell lineage-specific deletion of *Hdac3* (derived with the use if *Zbtb-Cre*) results in selective impairment in NKT1 cell development. The selective absence of HDAC3 in NKT cells resulted from reduced autophagy (193–195)—a cytoplasm recycling process essential to T and NKT cell development —and decreased GLUT1, CD71, and CD98 nutrient receptor expression (196). Moreover, the anti-tumor effect of αGalCer (109) is potentially mediated by IFNγ- and TNFα-producing NKT1 cells.

MOUSE NKT2 CELLS express the CD4 co-receptor. NKT2 cell actiation results in a Th2-like cytokine and chemokine response. This subset is enriched in mouse lungs and the intestine (152). IL-13 and IL-4 as well as CCL17, CCL22, CCL10/CCL6, and eosinophil chemotactic factor-L secreted by activated NKT2 cells may mediate airway hyperresponsiveness (151, 197–200). This Th2-type response recruits Mϕs, eosinophils, neutrophils, and lymphocytes into the lungs to incite tissue damage (197). Coincidently, in BALB/c mouse that is sensitive to airway hyperresponsiveness, NKT2 cells predominate (152).

NKT cells constitutively express *Il4* and *Ifng* transcripts. This constitutively expressed cytokine genes may explain the rapid NKT cell response to agonistic stimulation *in vivo* (201). Epigenetic changes in the two cytokine genes control their transcription. For example, the conserved non-coding sequence (CNS) 2 located downstream of the mouse *Il4* locus is constitutively active in NKT cells, which thereby constitutively transcribe the *Il4* gene. CNS 2 activity depends on NOTCH and Rbp-j (recombination signal binding protein for immunoglobulin kappa J region)—a transcriptional regulator of NOTCH signaling. Hence, DP thymocyte-specific deletion of *Rbp-j* abolished CNS 2 activity and the ability to transcribe *Il4* (202).

A similar epigenetic control of the human *Ifng* locus using CNS-30 and CNS +18–20 transcribes the *Ifng* locus in NKT cells (203, 204). Consistent with this finding, NKT cells showed acetylated histone 4 marks upstream and downstream of the *Ifng* coding region only when activated by weak (self agonists) or strong signals (phorbolmyristate acetate + ionomycin) but not in resting NKT cells. Furthermore, NKT cells rested after stimulation returned the *Ifng* locus to an unmarked state (205). H4 acetylation occurs at CNS +18–20, a site essential for human *Ifng* transcription in NKT cells and conserved within the mouse *Ifng* locus (203, 205). These findings notwithstanding, it is unclear whether human NKT cells constitutively transcribe the *Ifng* locus and how mouse NKT cells constitutively transcribe its *Ifng* locus.

MOUSE NKT17 CELLS do not express CD4 or CD8 co-receptors. They are enriched in the lungs, skin, and peripheral lymph nodes, and are poorly represented in the spleen and liver (206–208). These cells require IL-7, not IL-15, for survival (151, 209). The development of NKT17 cells also requires mTORC2 signaling and the transcription factors Runx1 and NKAP (168, 210–213). Thus, NKT cell-specific *Runx1* deletion results in decreased IL-7Rα, BATF, and c-Maf expression against the backdrop of increased Lef and Bcl11b expression (211). On the other hand, how NKAP controls NKT17 cell development is not understood, but appears not to require mTOR, IL-7, and TGF-β signaling (210).

Akin to Th17 cells, NKT17 cells constitutively express RORγt (206), rapidly produce IL-17A in response to certain bacterial infections, and induce airway neutrophilia when challenged with synthetic glycolipid or LPS (37, 206, 214). NKT17 cells may contribute to ozone-induced airway hypersensitivity (215), the development of experimental autoimmune encephalomyelitis (214), and the pathogenesis of acute hepatitis in mice (216).

MOUSE NKT10 CELLS, the PLZF-independent subset (154), are found in low frequency in unchallenged mice and in human peripheral blood mononuclear cells (PBMCs). Upon re-activation, NKT10 cells that previously responded to αGalCer *in vivo*, secrete IL-10 (155). IL-10 produced by activated NKT10 is thought to maintain immune-privilege sites. This NKT cell subset may also control Treg cell functions in adipose tissues (154).

Mouse NKT cells can provide cognate (lipid antigens) or non-cognate (protein antigens) help to B cells and regulate antibody responses (89, 90, 92, 217, 218). Upon immunization with αGalCer a subset of NKT cells acquire a phenotype similar to T follicular helper T cells (Tfh) referred to as NKT follicular helper (NKTfh) cells (218–220). NKTfh are characterized by the expression of CXCR5, ICOS, PD1, Bcl6, and BTLA. Their development is dependent on same factors that drive Tfh development (219). NKTfh cells induce rapid production of germinal centers through IL-21 production that yields detectable levels of antigen-specific IgG (91, 219, 220). Nonetheless, NKTfh cell-induced antibody responses are short-lived and inferior to Tfh cell-induced responses (91, 219, 220). NKTfh cells may play a role in antibody responses against human pathogens such as *Borrelia hermsii, Streptococcus pneumoniae*, and *Plasmodium falciparum* (91, 219, 220). NKTfh and Tfh cells can act synergistically to induce robust antigen-specific antibody responses underscoring the use of αGalCer as a vaccine adjuvant (218).

Human NKT cell responses are as diverse as those of mouse (221), yet NKT cell subsets have not been formalized in humans. Functional dichotomy has been reported in human CD4^+^ and DN NKT cell subsets: activated human CD4^+^ NKT cells secrete IL-4. A pathological role has been attributed to human CD4^+^ NKT cells, which accumulate in the lungs of chronic asthmatic patients and produce IL-4 and IL-13 (222). Hence, human CD4^+^ NKT cell resembles the mouse NKT2 cell subset. On the other hand, the activated DN NKT cells secrete IFN-γ and TNF-α. Furthermore, both CD4 and DN human NKT cell subsets upregulate perforin in the presence of inflammatory signals. The DN NKT cells also upregulate NKG2D expression, which together with perforin may mediate cytotoxicity against infected cells and cancer cells (223, 224). These functions of human NKT cells resemble those of mouse NKT1 cells. Activated human NKT cells can also secrete IL-17 (221), suggesting the presence of an NKT17-like subset.

In summary, mouse NKT cells divide labor into four subsets. Global and single cell transcriptome analyzes demonstrated that the thymic NKT1, NKT2, and NKT17 cells were distinct subsets (156, 225). Even though not formalized, human NKT cells also have the potential to mirror mouse NKT cell subsets, but this requires further investigation. That the tissue environment plays a role in the differentiation of NKT cell subsets is supported by the finding that NKT17 differentiation required mammalian target of rapamycin complex-2 (213) or is suppressed by Tet enzymes that modify 5-methylcytosine in DNA by controlling the expression of Tbet and ThPOK transcription factors (226). Another study using somatic cell nuclear transfer to generate mice with monoclonal NKT cell populations demonstrated that tissue homing pattern, and, to a lesser extent, TCR avidity governed NKT cell subset differentiation (208). That NKT1, NKT2, and NKT17 cells differentiated within peripheral tissues of each of the three monoclonal mouse lines, derived from somatic cell nuclear transfer, suggests that the subsets are perhaps NKT cell “reaktionsnorm [German for reaction norm or norm of reaction; Woltereck 1909 cited in Ref. (227)]” induced by the tissue-specific environment, potentially by local cytokine/chemokine milieu in conjunction with the host microbiota.

## NKT Cell Frequency Variation

An intriguing property of NKT cells is their frequency variation observed in lymphoid tissues of different inbred strains of similar age: low in 129 and NOD, intermediate in C57Bl/6, and high in BALB/c, CBA, and DBA/2 mice (152, 153, 228–230). Likewise, NKT cells show striking frequency variation that can range from as little as 0.001% to 5–10% within human PBMCs (221, 231, 232).

Mice show inter-strain variation in thymic NKT cell subset numbers (152). C57Bl/6 mice have high proportion of NKT1 cells and low frequency of NKT2 cells, whereas BALB/c have high frequency of NKT2 and NKT17 suggesting an inverse correlation between frequency of NKT1 cells versus NKT2 cells and mouse strains. Curiously, mouse strains that have a high frequency of NKT2 cells (BALB/c, CBA, and DBA/2) showed high numbers of eomesodermin-expressing memory-like CD8^+^ thymocytes (152) which was attributed to the steady-state production of IL-4 by the expanded NKT2 population in these mice. In an effort to understand whether genetic polymorphisms between mouse strains controlled NKT cell frequency, recombinant inbred and co-isogenic strains begotten from NOD (low NKT cell frequency) X C57Bl/10 (intermediate NKT cell frequency) crosses were analyzed. The outcomes of several such studies indicated that NKT cell frequency segregated with the genetic background of the mouse (153, 229, 230). Whereas this outcome suggests that NKT cell frequency is under genetic control, whether this control is direct or indirect remains to be ascertained.

## Developmental Symbiosis: Gut Microbiota-Dependent Peripheral NKT Cell Frequency and NKT Cell Control Over Gut Microbiota

NKT cells surveil barrier mucosae such as that of the small and large intestine (233, 234). The number, phenotype, and functional maturation of NKT cells in the gut epithelium and lamina propria are controlled by neonatal colonization of the gut by bacterial symbionts. Thus, germ-free (GF) mice have high numbers of NKT cells in the gut epithelium and lamina propria that are immature and, hence, hypo-responsive to αGalCer (233). Curiously, reconstitution of young, but not adult mouse gut by bacteria that biosynthesise αGalCer or related compounds reverses the hypo-responsiveness of NKT cells found in GF intestinal mucosae (234). Similarly, GF mice also harbor high hepatic and pulmonary, but not thymic and splenic NKT cell frequencies (234). Additional evidence implicates the CXCR6 ligand CXCL16, whose expression is under the control of gut microbiota, in regulating gut NKT cell frequency and maturation (234, 235). Furthermore, αGalCer compounds (see Table 1) synthesized by the bacterial symbiont *Bacteriodes fragilis*, exert either an inhibitory effect preventing proliferation, or are stimulatory on developing NKT cells (26, 27). As the gut microbiota varies between individuals of different genetic, ethnic, and geographic backgrounds (236), the above findings in mice suggest the intriguing possibility that the human symbionts may impart an epistatic control over human NKT cell frequency and maturation as well. Because the frequency and functional status are environmentally controlled even though the genotype of the differentiating NKT cells remains the same, NKT cell frequency and proper maturation are potentially polyphenic (227, 237) properties.

Early-life microbial ecology has implications for health. Thus, GF mice are prone to severe airway hypersensitivity and dextran sodium sulfate-induced colitis (233–235). The latter phenotype is obviated by the interaction of NKT cells with *B. fragilis*-derived glycosphingolipid(s) during early life (26). Not surprisingly, NKT cells can, in turn, control gut microbial ecology and gut physiology (238). Whether similar reciprocal interactions between NKT cells and the gut microbiota occur in humans currently remains unknown.

Microbial ecology has emerged as an important deterministic factor in the outcome of chemotherapy, radiation therapy, and immunotherapy against cancers (239). NKT cells have been targeted in the clinic for immunotherapy (see Implications for Cancer Immunotherapy), but how each of these therapies impact NKT cells is not known. It is noteworthy that a fraction of NKT cells are radiation resistant (130). This feature can be exploited for NKT cell-targeted immunotheraphy against lymphomas and leukemias. Clinical trials have shown that the outcome of NKT cell-targeted immunotherapy varied between recipients (103, 104). Hence, what roles the gut microbiota played in the outcome is worthy of investigation. So also, considering that NKT cells can impact microbial ecology (238), what roles NKT cells play in tumorigenesis and metastasis are also worthy of investigation. Insights into how the microbial community assembles and forms the host–symbiont ecosystem will facilitate an essential understanding of the molecular underpinnings that govern reciprocal interactions between the host and its internal ecosystem. These new insights can, in turn, impact the way by which new cancer therapies are designed, developed, and refined.

### Evolution of Type I NKT Cells

… the struggle against diseases, and especially infectious diseases, has been a very important evolutionary agent and that some of its results have been unlike those of the struggle for life … [(240) within a collection of papers in genetics by Haldane (241)].

Comparative vertebrate genomics, enabled by recent advances in whole-genome sequencing, have revealed molecular signatures of selection upon genes that control many biologic functions, including immune responses. Hence, pathobionts can apply immense selection pressure and play significant roles in the evolution of immune response genes and cells. As early-life symbionts can impact health, microbial ecology may also play roles in the evolution of the immune response genes and cells.

**Figure 6:**
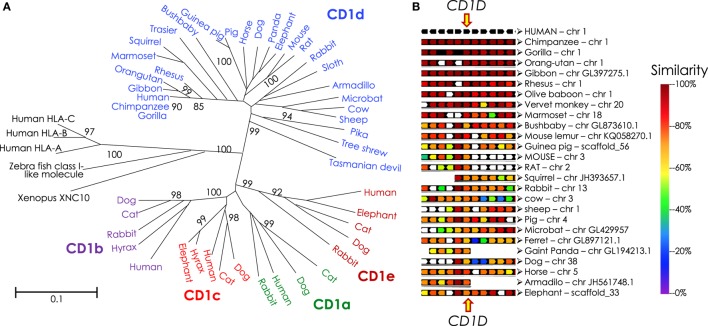
Phylogenetic tree of CD1 and syntenic relationship of eutherian *Cd1d*. **(A)** Protein sequences of α1-α2 domains of mammalian and reptilian CD1, human and zebra fish MHC class I and *Xenopus* XNC10 were retrieved from NCBI using PSI-BLAST. Sequences with *E*-value of ≤0.05 were considered mostly similar to the human CD1D query sequence; significant bootstrap values are indicated for critical nodes. Sequences were aligned using ClustalW. Phylogenetic analysis (pairwise deletion, bootstrap: *n* = 1,000) was performed to construct the optimal tree with the sum of branch length = 9.42589507. Neighbor-Joining method (245) was used to infer evolutionary history. An optimal tree with the sum of branch length = 9.42589507 is shown. p-distance method was used to compute evolutionary distances (246, 247). The tree is drawn to scale, with branch length units the same as those of the evolutionary distances used to infer the phylogenetic tree. A total of 51 sequences and 210 positions were included in the final dataset. Evolutionary analyses were performed using MEGA6 (248). Mammalian species names according to Ref. (249), and the Order/Family to which they belong according to Ref. (250, 251) as well as homology to human CD1D are as follows: northern brown bandicoot (*Isoodon macrourus*), Peramelemorphia/Peramelidae, 50%; Tasmanian devil (*Sarcophilus harrisii*), Marsupialia/Dasyuridae, 42%; Hoffmann’s two-toed sloth (*Choloepus hoffmanni*), Pilosa (Xenarthra)/Megalonychidae, missing various parts of the protein; nine-banded armadillo (*Dasypus novemcinctus*), Cingulata (Xenarthra)/Dasypodidae, 62%; tailless tenrec (*Echinops telfairi*), Afrosoricida (Insectivora)/Tenrecidae, missing N- and C-terminal ends of the protein; western European hedgehog (*Erinaceus europaeus*), Erinaceomorpha (Insectivora)/Erinaceidae; Eurasian shrew (*Sorex araneus*), Soricomorpha (Insectivora)/Soricidae, has only 181 aa, missing N- and C-terminal ends of the protein; northern tree Shrew (*Tupaia belangeri*), Scandentia/Tupiidae, 68%, missing leader sequence; large flying fox (*Pteropus vampyrus*): Chiroptera/Petropodidae; little brown bat (*Myotis lucifugus*), Chiroptera/Vespertilionidae 62%; dog (*Canis lupus familiaris*), Carnivora/Canidae, 57%, missing initiator methionine; giant panda (*Ailuropoda melanoleuca*), Carnivora/Ursidae, 60%, missing initiator methionine; ferret (*Mustela putorius furo*), Carnivora/Mustelidae, 60%, missing leader sequence; cat (*Felis catus*), Carnivora/Filidae; bottlenose dolphin (*Tursiops truncatus*), Cetacea/Delphinidae; African bush elephant (*Loxodonta africana*) Probosidea/Eliphantidae 65%; horse (*Equus caballus*), Perissodactyla/Equidae, 73%; rock hyrax (*Procavia capensis*), Hyracoidea/Procavidae, 64%; wild boar (*Sus scrofa*), Artiodactyla/Suidae, 65%; alpaca—Andean paca (*Vicugna pacos*), Artiodactyla/Camelidae; cow (*Bos taurus*), Artiodactyla/Bovidae, 65%, valine substitution of initiator methionine; squirrel (*Ictidomys tridecemlineatus*), Rodentia/Sciuridae, 64%, missing last 12 aa including the recycling motif; Ord’s kangaroo rat (*Dipodomys ordii*): Rodentia/Heteromyidae; brown rat (*Rattus norvegicus*), Rodentia/Muridae; 64%; mouse (*Mus musculus domesticus*), Rodentia/Muridae, 61%; guinea pig (*Cavia porcellus*), Rodentia/Cavidae, 65%; American pika (*Ochotona princeps*), Logomorpha/Ochotonidae, 64%, missing last 17 aa including the recycling motif; European rabbit (*Oryctolagus cuniculus*), Logomorpha/Leporidae, 67%; gray mouse lemur (*Microcebus murinus*), Primates/Cheirogaleidae, 78%; small-eared galago—a bushbaby (*Otolemur garnettii*), Primates/Galagidae (Loridae), 71%, missing last 31 aa including the recycling motif; Philippine tarsier (*Tarsius syrichta)*, Primates/Tarsiidae, 71%; white-tufted-ear marmoset (*Callithrix jacchus*), Primates/Callitrichidae, 83%; rhesus monkey/macaque (*Macaca mulatta*), Primates/Cercopithecidae, 88%; northern white-cheeked gibbon (*Nomascus leucogenys*), Primates/Hylobatidae, 95%; western gorilla (*Gorilla gorilla gorilla*), Primates/Hominidae, 99%; Sumatran orang utan (*Pongo abelii*), Primates/Hominidae, 96%; chimpanzee (*Pan troglodytes*), Primates/Hominidae, 98%; human (*Homo sapiens*), Primates/Hominidae. **(B)** Syntenic map was drawn using Genomicus v89.01 Phyloview (252). Human CD1D gene was used as reference gene and species, respectively, Eutheria as the root species. Upstream of CD1D gene are KIRREL, CD5L, FCRL1, FCRL2, and FCRL3 genes, and downstream from CD1D are CD1A, CD1C, CD1B, CD1E, and ORIOT2 genes in the syntenic map.

The NKTCR engages its ligand, CD1d-lipid co-complex, with conserved germline-encoded residues in four-to-five of the six complementarity-determining regions of the combined TCR α- and β-chains (242). Hence, phylogenetic studies of genes that encode CD1 molecules and the invariant NKTCR α-chain can reveal the origin and evolution of NKT cells. A recent phylogenomic analysis revealed that the *Cd1* gene is an amniote innovation that evolved in the Mesozoic reptiles and was retained in the extant anapsid (green anole lizard *Anolis carolinensis*) and synapsid (Siamese crocodile *Crocodylus siamensis* and Chinese alligator *Alligator sinensis*) reptilians (243). *Cd1* genes diversified in mammals, wherein evolved the *Cd1d* gene that encodes the lipid agonist presenting molecule that controls the functions of NKT cells in eutherians (of placental mammals; Figure 6) (244). Curiously however, the reptilian *Cd1* gene has no orthology with avian or mammalian *Cd1* genes (243), suggesting that *Cd1* genes may have emerged multiple times during amniote evolution. Or alternatively, *Cd1* genes may have evolved rapidly and diverged substantially from the reptilian form within extinct synapsid and mammal-like reptiles prior to stabilization within eutherian species. The latter view is supported by the finding that egg-laying monotremes such as platypuses and echidnas do not have *Cd1* genes while a *CD1d*-like gene exists in a few metatherian (of marsupial mammals) species such as the opossum.

A phylogenetic analysis of TRAV10 (encoding the human Vα24 gene segment) or TRAV11 (encoding the mouse Vα14 gene segment) and TRAJ18 (encoding the Jα18 gene segment) revealed that gene elements related to TRAV10/11 and TRAJ18 were found only in placental mammals (244). This finding suggests that NKT cells are a eutherian innovation. As the host–gut microbiota controls NKT cell terminal functional differentiation and NKT cells impact gut microbial ecology, it is postulated that placental development, sudden perinatal exposure to maternal and environmental microbiota, and lactation may have contributed to the evolution of CD1d-restricted type I NKT cells.

### A Final Analysis: Under the Spell of PLZF and Host Microbial Ecology, a Curious Case for a “Limbic Immune System!”

The foregoing discusses recent advances in developmental biology of NKT cells and the environmental context in which it develops, matures and differentiates. A final section discusses their evolutionary path and how developmental biology and ecology may have contributed to this unique developmental plan. In addition, how the eco-evo-devo perspective on NKT cells may contribute to cancer immunotherapy is touched upon. Finally, areas that will benefit from further investigation are also pin pointed in their respective sections. Summarily, such areas include, (a) what early events specify NKT cell lineage commitment and turn on the unique lineage-specific GRN?; (b) what signals do symbionts relay to developing NKT cells to specify physiologic functions?; (c) in turn, what signals do NKT cells relay to the microbial community in the gut, and potentially to the microbionts in skin and lungs, to ensure physiologic community assembly, structure, and organization in early, young, and adult life?; (d) what tissue environmental signals underlie NKT cell subset differentiation?; (e) can radiation resistance of NKT cells be used in cancer immunotherapy?; and (f) what NKT cell intrinsic and environmental signals have retained NKT cells in certain mammalian species but not in others?

As a final note to the devo-eco-evo synthesis, we observed that the unique behavior of a group of innate-like T lymphocytes and innate lymphoid cells (ILCs) are under the control of PLZF (253–255). These include γδ T cells, NKT cells, MAIT cells, and certain ILCs. In addition, the development (MAIT cells, and potentially γδ T cells) and functional differentiation (NKT cells, MAIT cells, and ILCs) of these cells are determined by gut and potentially other barrier (skin and lungs) symbionts. As these immune cells, all of lymphoid origin, function at the edge (limbus in Latin) of the innate and adaptive immune systems, a proposal to group them into the “limbic immune system” is made here. Curiously, γδ T, NK, and NKT cells localize to the inter-follicular region of the lymph nodes, straddling the cells of the innate and adaptive immune systems (256). By virtue of their physiologic functions, other tissue-restricted innate-like lymphocytes, such as CD8αα innate-type lymphocytes (257) as well as B1 cells and NK cells (258), can be included in the “limbic immune system” even though their development and function may not be controlled by PLZF or the microbiota. In other words, the “limbic immune system” is anglicized Latin for the “inbetweeners” (259) and, hence, synonymous with it.

## Author Contributions

All authors wrote and edited the manuscript.

## Conflict of Interest Statement

The authors declare that the research was conducted in the absence of any commercial or financial relationships that could be construed as a potential conflict of interest.
